# Hydrogen Bonding Interaction between Atmospheric Gaseous Amides and Methanol

**DOI:** 10.3390/ijms18010004

**Published:** 2016-12-30

**Authors:** Hailiang Zhao, Shanshan Tang, Xiang Xu, Lin Du

**Affiliations:** 1 Environment Research Institute, Shandong University, Shanda South Road 27, Jinan 250100, Shandong, China; zhaohl0211@sina.com (H.Z.); tangshanshan9210@sina.com (S.T.); 2 College of Chemistry and Pharmaceutical Sciences, Qingdao Agricultural University, Changcheng Road 700, Qingdao 266109, Shandong, China; xuxiang2016@sina.cn

**Keywords:** hydrogen bond, molecular complexes, energy decomposition analysis

## Abstract

Amides are important atmospheric organic–nitrogen compounds. Hydrogen bonded complexes of methanol (MeOH) with amides (formamide, *N*-methylformamide, *N*,*N*-dimethylformamide, acetamide, *N*-methylacetamide and *N*,*N*-dimethylacetamide) have been investigated. The carbonyl oxygen of the amides behaves as a hydrogen bond acceptor and the NH group of the amides acts as a hydrogen bond donor. The dominant hydrogen bonding interaction occurs between the carbonyl oxygen and the OH group of methanol as well as the interaction between the NH group of amides and the oxygen of methanol. However, the hydrogen bonds between the CH group and the carbonyl oxygen or the oxygen of methanol are also important for the overall stability of the complexes. Comparable red shifts of the C=O, NH- and OH-stretching transitions were found in these MeOH–amide complexes with considerable intensity enhancement. Topological analysis shows that the electron density at the bond critical points of the complexes fall in the range of hydrogen bonding criteria, and the Laplacian of charge density of the O–H∙∙∙O hydrogen bond slightly exceeds the upper value of the Laplacian criteria. The energy decomposition analysis further suggests that the hydrogen bonding interaction energies can be mainly attributed to the electrostatic, exchange and dispersion components.

## 1. Introduction

Amides have attracted great attention due to their importance to N–H∙∙∙O hydrogen bonding interaction in determining the structures and properties of biological systems, such as the interactions between protein and polypeptides [1,2,3]. Amide is a simplified example of peptide linkage, which allows studying both hydrophobic and hydrophilic interactions. On the other hand, amides have been widely observed in the atmosphere [4]. Amides are emitted from a variety of natural and anthropogenic sources including agriculture, biomass burning, animal husbandry, cooking, synthetic leather, carbon capture, and other industrial processes [4,5,6]. For example, formamide (FA, HC(O)NH_2_) is a high production volume chemical (HPV) with an annual global production of several hundred thousand tons [7]. FA was detected in the emissions from an industrial carbon capture facility via degradation of mono-ethanolamine, using proton transfer reaction time-of-flight mass spectrometry (PTR-ToF-MS) [5]. *N*,*N*-dimethylformamide (DMF) was observed near a municipal incinerator, waste collection center and sewage treatment plant with a concentration of 1.2–14.2 μg·m^−3^ [8]. Moreover, amides can also be formed by the degradation of amines [6]. Amide in the atmosphere can react with OH radicals, and lead to gaseous degradation products and formation of secondary organic aerosols [7,9].

New particle formation (NPF) is an important mechanism to form atmospheric aerosols [10]. NPF has been estimated to account for roughly half the global budget of the atmosphere’s cloud condensation nuclei (CCN), which influences cloud properties and Earth’s energy balance [11]. At the molecular level, molecular complexes and pre-nucleation clusters are at the initial stage of NPF whose strength determines the thermodynamic stability of these complexes. Hydrogen bonding interaction is one of the driving forces for the formation of atmospheric molecular complexes. The composition in the initial nucleation clusters is very critical [12,13,14,15], but the species involved in the atmospheric nucleation are still largely unknown [16,17]. It is well known that trace atmospheric organic species other than H_2_SO_4_ and H_2_O are involved in nucleation [18]. Atmospheric methanol (MeOH) is one of the most abundant organic compounds in the atmosphere [12,19,20]. Fifty percent of the measured oxygenated volatile organic compounds (OVOCs) in Zürich were alcohols [20]. Furthermore, 11.9% of the top 20 OVOC (by mass) emissions from anthropogenic sources (in the UK) in 2010 was MeOH [19]. Due to the importance of MeOH in the atmosphere, many studies have been carried out to investigate the initial stages of the atmospheric aerosol nucleation involving MeOH [21]. A matrix isolation spectroscopic method and theoretical studies were carried out to investigate the hydrogen bonding in the H_2_SO_4_/MeOH/H_2_O system [21]. The results revealed that the observed complexes may be precursors in the atmospheric sulfate and bisulfate cluster formation. Amine is an important compound for atmospheric nucleation [22,23]. Fourier transform infrared (FTIR) and density functional theory (DFT) studies have been carried out for the interactions between MeOH and dimethylamine/trimethylamine complexes [24]. The IR spectrum shows a characteristic red shift of the OH-stretching vibrational band of 301 and 333 cm^−1^ for MeOH–dimethylamine and MeOH–trimethylamine, respectively. The calculated binding energies for these two complexes were about −25~−27 kJ·mol^−1^ (B3LYP/aug-cc-pVTZ).

The impact of the molecular interactions between MeOH and amides in atmospheric clustering has been investigated in the present study. Theoretical calculations of complexes consisting of MeOH and the atmospheric common amides (formamide (FA), *N*-methylformamide (NMF), *N*,*N*-dimethylformamide (DMF), acetamide (AA), *N*-methylacetamide (NMA) and *N*,*N*-dimethylacetamide (DMA)) were carried out to determine the geometric parameters, binding energies and Gibbs free energies of formation. Atoms in molecules (AIM) analysis was used to investigate the electronic densities and the inter-molecular hydrogen bonding interactions in the complexes [25,26]. Generalized Kohn-Sham energy decomposition analysis (GKS-EDA) was performed to study the contribution of different components to total interaction energy [27].

## 2. Results and Discussion

### 2.1. Geometric Analysis

**Figure 1:**
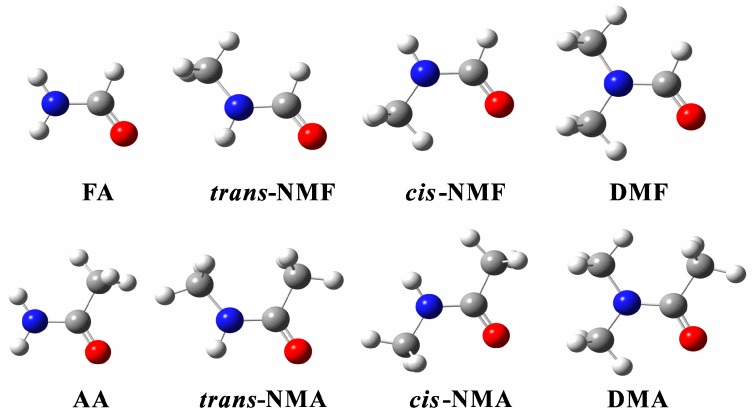
The stable structures of the monomers obtained at the B3LYP-D3/aug-cc-pVTZ level; FA: formamide; NMF: *N*-methylformamide; DMF: *N*,*N*-dimethylformamide; AA: acetamide; NMA: *N*-methylacetamide; DMA: *N*,*N*-dimethylacetamide.

The optimized structures of the amide monomers at the B3LYP-D3/aug-cc-pVTZ level are shown in Figure 1. Both NMF and NMA have two isomers, where *cis* and *trans* refer to the orientation of the C=O and N–H bonds with respect to the C–N bond. Previous studies demonstrated that the *trans*-form was more stable in the gas phase [28,29,30], which is consistent with our study: *trans*-NMF > *cis*-NMF by 4.8 kJ·mol^−1^ (B3LYP-D3/aug-cc-pVTZ, corrected with ZPVE) and *trans*-NMA > *cis*-NMA by 9.7 kJ·mol^−1^ (B3LYP-D3/aug-cc-pVTZ, corrected with ZPVE).

**Figure 2:**
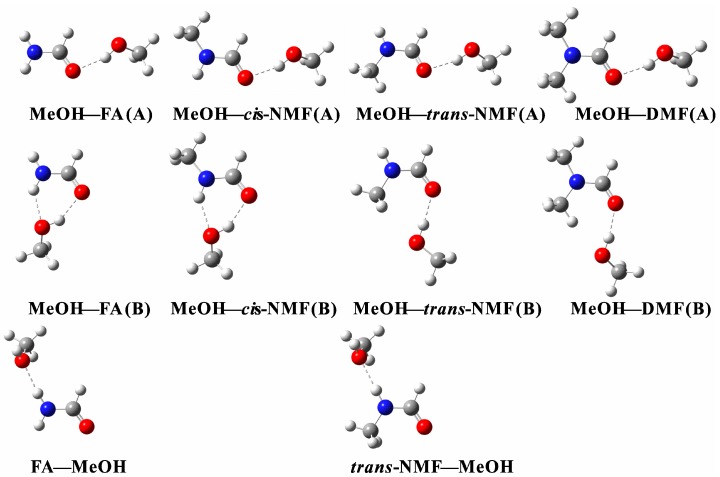
The stable structures of the MeOH–formamide and formamide–MeOH complexes obtained at the B3LYP-D3/aug-cc-pVTZ level; The O–H∙∙∙O and N–H∙∙∙O hydrogen bonds are presented by the dotted lines; FA: formamide; NMF: *N*-methylformamide; DMF: *N*,*N*-dimethylformamide; MeOH: methanol.

**Figure 3:**
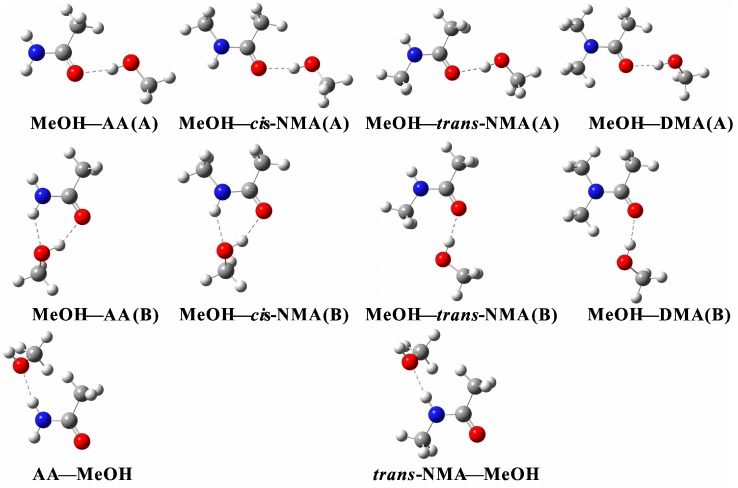
The stable structures of the MeOH–acetamide and acetamide–MeOH complexes obtained at the B3LYP-D3/aug-cc-pVTZ level; The O–H∙∙∙O and N–H∙∙∙O hydrogen bonds are presented by the dotted lines; AA: acetamide; NMA: *N*-methylacetamide; DMA: *N*,*N*-dimethylacetamide; MeOH: methanol.

Several orientations of MeOH with respect to the carbonyl oxygen were examined. There are two possible types of hydrogen bonding interactions in the complexes, O–H∙∙∙O=C and N–H∙∙∙O. The MeOH–amide (A) structures (Figure 2 and Figure 3) are the ones in which MeOH approaches the carbonyl oxygen from the opposite side of the N atom. The main interaction occurs between the OH group of MeOH and the carbonyl oxygen of the amides to form the O–H∙∙∙O=C hydrogen bonds. When MeOH moves towards the carbonyl oxygen from the same side of nitrogen, the structure is noted as (B) in Figure 2 and Figure 3. Interestingly, the MeOH–FA (B), MeOH–AA (B), MeOH–*cis*-NMF (B), MeOH–*cis*-NMA (B) complexes form six-membered cyclic ring structures via the O–H∙∙∙O=C and N–H∙∙∙O hydrogen bonds. Meanwhile, the NH groups in AA, FA, *trans*-NMA and *trans*-NMF act as hydrogen bond donors while MeOH is the hydrogen bond acceptor to form N–H∙∙∙O hydrogen bonded complexes, and they are noted as amide–MeOH. Amide nitrogen is generally a poor nucleophile because of the amide resonance. The conjugation with the electron-withdrawing carbonyl group reduces the electron density of the nitrogen atom. In addition, the steric hindrance between terminal methyl groups also prevents the N atom from being a proton acceptor.

Selected geometric parameters obtained at the B3LYP-D3/aug-cc-pVTZ level are listed in Table 1. The interactions between MeOH and amide are called resonance-assisted hydrogen bonding (RAHB), where they involve a category of homo-nuclear hydrogen bonds (–O–H∙∙∙O=) where two oxygen atoms are inter-connected by a system of *π*-conjugated double bonds [31,32,33,34]. The contact distance *d*(O---O) between the two oxygen atoms in the hydrogen bond (–O–H∙∙∙O=) has been employed as an indicator of the hydrogen bond strength [31]. The contact distances in the MeOH–amide (A) and (B) structures are in the range of 2.76–2.83 Å, and belong to medium or weak hydrogen bonds.

Proton transfer may occur between interacting molecules which leads to additional cluster stabilization. An ion pair can be formed via proton transfer from an acid moiety (e.g., H_2_SO_4_) to a base moiety (e.g., NH_3_, amine). In the H_2_SO_4_–DMA complex, the OH bond of H_2_SO_4_ was elongated by 0.710 Å (PW91PW91/6-311++G(2d, 2p)) upon complexation [35]. Due to the higher proton affinity of amines, the proton was transferred from oxygen in H_2_SO_4_ to nitrogen in DMA. However, proton transfer did not occur in succinic acid–DMA–(H_2_O)_0–3_, where the OH bond lengths were only elongated by 0.108–0.244 Å (PW91PW91/6-311++G(2d, 2p)) [35]. In this study, the changes in the OH bond length of MeOH upon complexation (Δr(OH) in Table 1) vary from 0.010 to 0.017 Å in the MeOH–amide (A) and (B) complexes, however, the changes in the NH bond length of amides upon complexation (Δr(NH) in Table 1) are much smaller (<0.009 Å) in the MeOH–amide (B) and amide–MeOH complexes. The relative small changes of the OH or NH bond upon complexation clearly reveal that there is no proton transfer in the hydrogen bonding interactions between MeOH and amides.

The formation of a strong hydrogen bond between the OH group of MeOH and the carbonyl group leads to an elongation of the C=O bond (Δr(C=O) in Table 1), which is about 0.007–0.013 Å in the MeOH–amide (A) and (B) complexes. In an early study on hydrogen bonded complexes between lactones with 3,4-dinitrophenol, the C=O bonds were calculated to be elongated by 0.011–0.017 Å (B3LYP/6-311+G*) [36]. In addition, the C=O bonds also marginally increase by about 0.003–0.004 Å in the amide–MeOH complexes. The hydrogen bond angles in the four cyclic structures, namely, MeOH–FA (B), MeOH–AA (B), MeOH–*cis*-NMF (B), MeOH–*cis*-NMA (B), deviate within 30° from the ideal linear orientation. The hydrogen bond angles of the remaining structures vary from 158° to 177°.

Furthermore, it is worth noting that the unsubstituted amides are less basic than the methyl substituted ones. The inductive effect of the methyl group enhances the basicity of the carbonyl group [36,37]. It can be seen that the changes in the OH bond of MeOH upon complexation are increased from FA to AA, NMF to NMA, DMF to DMA, FA to NMF, and AA to NMA (Table 1). DMF and DMA are substituted with two methyl groups, but the OH bond is marginally changed as compared with the mono-substituted NMF and NMA. When amide acts as a hydrogen bond donor, the changes of the NH bond upon complexation is decreased from FA to AA, *trans*-NMF to *trans*-NMA, but increased from FA to *trans*-NMF, AA to *trans*-NMA. Therefore, the substitution on the carbonyl group can enhance the strength of the hydrogen bond donor on amide, but the substitution on the amine group may weaken the strength of the hydrogen bond donor on amide.

### 2.2. Interaction Energy

The thermal properties were calculated at the B3LYP-D3/aug-cc-pVTZ level at 298.15 K and 1 atm, and the binding energy (*BE*), enthalpy of formation (Δ$H_{298K}^{\theta }$) and Gibbs free energy of formation (Δ$G_{298K}^{\theta }$) are summarized in Table 2. The values computed with the B3LYP-D3 functional have been found to give reasonable hydrogen bonding interaction energies [38,39,40,41]. The *BEs* of the MeOH complexes are in the range of −21 to −39 kJ·mol^−1^. In an early study, the *BEs* of 3,4-dinitrophenol (DNP) with lactones (α-acetolactone, β-propiolactone, γ-butyrolactone, δ-valerolactone, ε-caprolactone) were calculated to be −22.8 to −47.4 kJ·mol^−1^ (B3LYP/6-311++G**) [36]. The calculated *BEs* for the MeOH–dimethylamine and MeOH–trimethylamine complexes were calculated to be −25~−27 kJ·mol^−1^ (B3LYP/aug-cc-pVTZ) [24]. In general, the MeOH–amide (B) complexes are more stable than their corresponding MeOH–amide (A) and amide–MeOH complexes. However, the MeOH–amide (A) structures of *trans*-NMA, DMF and DMA are very closed to their corresponding MeOH–amide (B) structures in terms of *BEs*, therefore, both should be present as an equilibrium mixture in the gas phase.

The MeOH–*cis*-NMF (B) and MeOH–*cis*-NMA (B) complexes are relatively more stable than their corresponding MeOH–*trans*-amide (B) conformers, although the *trans*-NMF and *trans*-NMA are much more stable than their *cis*-monomers. For the MeOH–NMF and MeOH–NMA complexes, the energy of *cis*- and *trans*- conformers are very close to each other.

### 2.3. OH-Stretching Vibrational Frequencies

IR spectroscopy has been considered as one of the most important methods to investigate hydrogen bonding interactions. The red shift (Δ$\tilde{\nu }$) between the free and hydrogen bonded OH-stretching transition is very useful in determining the relative strength of hydrogen bonding interaction [39,42]. The calculated C=O, NH- and OH-stretching fundamental transition wavenumbers and the red shifts upon complexation of the complexes at the B3LYP-D3/aug-cc-pVTZ level are summarized in Table 3.

The red shifts of the OH-stretching frequencies in the MeOH–amide (A) and (B) complexes were in the range of 147–320 cm^−1^ and the intensities were increased more than 19 times with respect to the MeOH monomer. The red shifts of the OH-stretching vibrational transition of the O–H∙∙∙O hydrogen bond upon complexation were reported to be 169 cm^−1^ (B3LYP-D3/aug-cc-pVTZ) for MeOH–*N-*nitrosodimethylamine (NDMA) [43]. For MeOH–dimethyl ether (DME), the calculated O–H∙∙∙O hydrogen bond frequency was 3659 cm^−1^ (B3LYP/aug-cc-pVTZ) with a red shift of 170 cm^−1^ and the corresponding intensity was increased by 18 times as compared with the monomer [44].

Both the AA and FA complexes have a NH_2_ group and the calculated IR spectra show larger red shifts of the symmetric NH-stretching transitions (66–131 cm^−1^) than those of their corresponding anti-symmetric NH-stretching transitions (28–53 cm^−1^). The enhancement of the intensities is less than 10 times with respect to the monomers. A similar trend was found in previous study on the hydrogen bonding interactions between H_2_O/MeOH with melamine (MA) [44]. MA has three NH_2_ groups, and the most stable structure was formed via O–H∙∙∙O and N–H∙∙∙O hydrogen bonds. The red shifts of the symmetric NH-stretching transitions were calculated to be 71–86 cm^−1^ (B3LYP/aug-cc-pVTZ) and values of the anti-symmetric NH-stretching transitions were 28–32 cm^−1^ (B3LYP/aug-cc-pVTZ). In contrast, the NH-stretching transitions in the MeOH–*cis*-NMF (B) and MeOH–*cis*-NMA (B) complexes are much more red shifted (149–60 cm^−1^) than those in the MeOH–*trans*-NMF and MeOH–*trans*-NMA complexes (35–95 cm^−1^). MeOH acts as a hydrogen bond donor and acceptor simultaneously in the former two complexes, which greatly enhances the electronegativity of the oxygen atom of MeOH. In addition, MeOH acts a hydrogen acceptor at the same time for both the N–H∙∙∙O and C–H∙∙∙O hydrogen bonds in the *trans*-NMF–MeOH and *trans*-NMA–MeOH complexes. Under such circumstances, the contribution of MeOH to each acceptor will be significantly reduced. As a result, the red shifts of the NH-stretching transitions are much smaller than those in the MeOH–*cis*-NMF (B) and MeOH–*cis*-NMA (B) complexes.

The calculated C=O stretching fundamental transition wavenumbers and the red shifts of the complexes are listed in Table 3. The C=O stretching vibrational transition of the O−H∙∙∙O=C bonded MeOH–amide complexes were calculated to be red shifted by 16–35 cm^−1^ with respect to the corresponding amide monomers. The red shifts were caused by electronic charge released from the C=O bond of amide to the OH of alcohol during the hydrogen bond formation [45]. In a previous study, comparable red shifts of the C=O stretching transition (21–34 cm^−1^) were observed between phenol derivatives and methyl acetate/methyl chloroacetate in carbon tetrachloride solution by FTIR spectroscopy [46]. Moreover, the C=O stretching bands were red shifted less than 40 cm^−1^ (B3LYP/6-311+G**) in the MeOH complexes with various cyclic ketones, lactones, lactams, and *N*-methyl lactams [37]. The C=O stretching vibrational transition of the O–H∙∙∙O=C bonded amide–MeOH complexes were calculated to be red shifted by 7–12 cm^−1^ with respect to the corresponding amide monomers.

### 2.4. Topological and GKS-EDA Analysis

**Figure 4:**
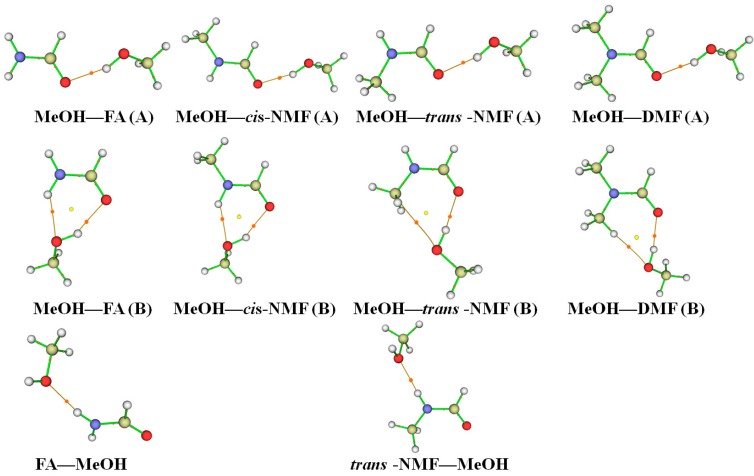
The Atoms in Molecules (AIM) plots of the MeOH–formamide and formamide–MeOH complexes obtained at the B3LYP-D3/aug-cc-pVTZ level; The bond critical points (BCPs) and ring critical points (RCPs) are presented by the ginger and yellow balls, respectively; FA: formamide; NMF: *N*-methylformamide; DMF: *N*,*N*-dimethylformamide; MeOH: methanol.

**Figure 5:**
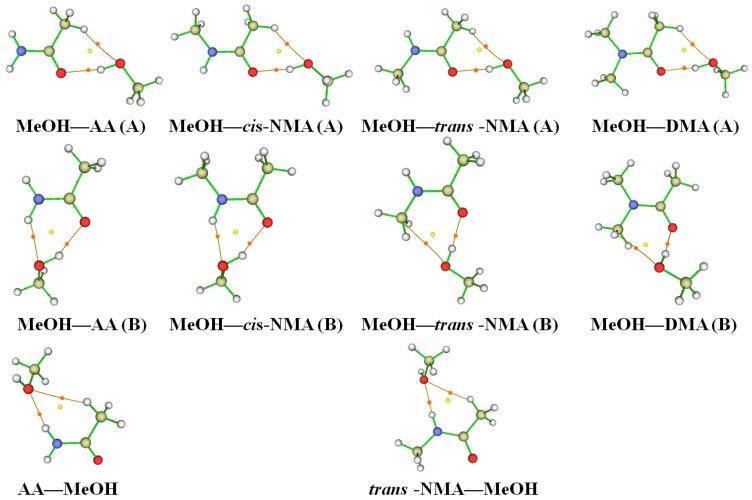
The Atoms in Molecules (AIM) plots of the MeOH–acetamide and acetamide–MeOH complexes obtained at the B3LYP-D3/aug-cc-pVTZ level. The bond critical points (BCPs) and ring critical points (RCPs) are presented by the ginger and yellow balls, respectively; AA: acetamide; NMA: *N*-methylacetamide; DMA: *N*,*N*-dimethylacetamide; MeOH: methanol.

Topological analyses by Markovian electron localization function (ELF) [47], delocalization behavior [48], and electron density *ρ*(r) using quantum theory of atoms in molecules (QTAIM) [25,26], are commonly used to analyze structure, bonding and chemical reactivity. In this study, AIM analysis was used to evaluate the hydrogen bond strength. AIM analysis was performed using the wave functions calculated at the B3LYP-D3/aug-cc-pVTZ level. The AIM plots of the complexes with bond critical points (BCPs), ring critical points (RCPs) and electron density paths are shown in Figure 4 and Figure 5. The AIM molecular graph shows the BCPs along the lines joining the OH (or NH) and O atom for the MeOH–amide and amide–MeOH complexes, which clearly prove the presence of hydrogen bonds. The topological parameters, including electron density *ρ*(r), Laplacian ∇^2^*ρ*(r) at the BCPs, and change in atomic charge Δ*q*(H) at the H atom with the B3LYP-D3/aug-cc-pVTZ method are listed in Table 4.

The C–H···O hydrogen bonds have been widely studied in recent literature, where the hydrogen bond donors were electronegative oxygen atoms of various functional groups [49,50]. From an energetic point of view, C–H···O hydrogen bonds are much weaker than the classical X–H···Y hydrogen bonds where the X and Y are electronegative atoms such as nitrogen, oxygen or sulfur. A typical feature for C–H···O hydrogen bonds are the blue shifts of the C–H vibrational fundamental transitions with lower transition intensities with respect to the monomer [51,52]. The C–H∙∙∙O hydrogen bonds are weak non-covalent interactions and they are referred to as cooperative hydrogen bonding interactions. The cooperative interactions play an important role in determining the structure and properties of materials [53,54].

According to the criteria proposed by Popelier, the electron density *ρ*(BCP) at the BCPs is in the range of 0.002–0.040 a.u. for a hydrogen bond [55,56]. The electron densities at the BCPs are in the ranges of 0.0226–0.0252, 0.0059–0.0095 and 0.0178–0.0204 a.u. for the O–H∙∙∙O, C–H∙∙∙O and N–H∙∙∙O hydrogen bonds, respectively. Similarly, the Laplacian of charge density ∇^2^*ρ*(BCP) of the complexes is in the ranges of 0.1254–0.1513, 0.0210–0.0412 and 0.0922–0.1052 a.u. for the O–H∙∙∙O, C–H∙∙∙O and N–H∙∙∙O hydrogen bonds, respectively. These values for the C–H∙∙∙O and N–H∙∙∙O hydrogen bonds are in the range of the Laplacian criteria (0.014–0.139 a.u.) for a hydrogen bond [55,56]. The values for the O–H∙∙∙O hydrogen bond are slightly larger than the upper value of the Laplacian criteria.

The distance between a BCP and an RCP has been used as a criteria to measure the structural stability of a hydrogen bond [57]. The MeOH–amide (A), MeOH–*trans*-NMA (B) and MeOH–DMA (B) complexes are formed by two hydrogen bonds: O–H∙∙∙O and C–H∙∙∙O. The distances between the BCP of the O–H∙∙∙O hydrogen bond and the RCP in the multi-membered rings are much larger (1.8371–2.0597 Å) than their corresponding distances between the BCP of the C–H∙∙∙O hydrogen bond and the RCP (0.7400–1.1806 Å). This shows that the O–H∙∙∙O hydrogen bonds are relatively more stable than the C–H∙∙∙O hydrogen bond in the MeOH–amide (A), MeOH–*trans*-NMA (B) and MeOH–DMA (B) complexes. The MeOH–FA/AA/*cis*-NMF/*cis*-NMA (B) complexes are formed by two hydrogen bonds: O–H∙∙∙O and N–H∙∙∙O. The distances between the BCP of the O–H∙∙∙O hydrogen bond and the RCP are slightly larger than those of N–H∙∙∙O. In the AA–MeOH and *trans*-NMA–MeOH complexes, the distances between the BCP and the RCP clearly indicate that the N–H∙∙∙O hydrogen bonds are much stronger than the C–H∙∙∙O hydrogen bond.

**Figure 6:**
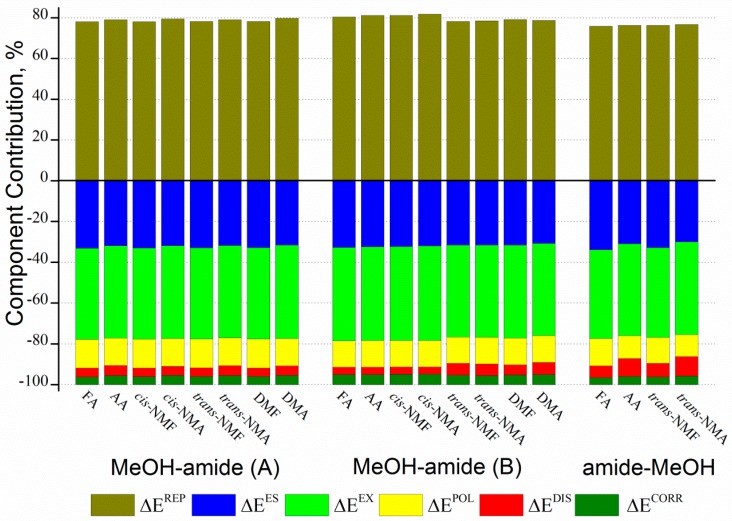
The six components’ contribution (%) to the total interaction energy in generalized Kohn-Sham energy decomposition analysis (GKS-EDA) at the B3LYP-D3/aug-cc-pVTZ level; Positive values are the repulsive component (repulsion energy (E^REP^)), and the negative values are the attractive components (electrostatic energy (E^ES^), exchange energy (E^EX^), polarization energy (E^POL^), correlation energy (E^CORR^) and Grimme’s dispersion energy (E^DISP^)); FA: formamide; NMF: *N*-methylformamide; DMF: *N*,*N*-dimethylformamide; AA: acetamide; NMA: *N*-methylacetamide; DMA: *N*,*N*-dimethylacetamide.

To get a better insight into the nature of hydrogen bonding interaction, it is important to decompose the non-covalent interaction into various “components” and to quantify their contributions to the binding energy. We have utilized Su’s generalized Kohn-Sham energy decomposition analysis (GKS-EDA) for energy decomposition analysis [58]. GKS-EDA method allows to fragment the total interaction energy (E^INT^) into electrostatic energy (E^ES^), exchange energy (E^EX^), repulsion energy (E^REP^), polarization energy (E^POL^), Grimme’s dispersion energy (E^DISP^) and correlation energy (E^CORR^). The total interaction energy along with the individual components were obtained using the GKS-EDA scheme at the B3LYP-D3/aug-cc-pVTZ level (Table 5). The attractive E^DISP^ term computed in the GKS-EDA method arises due to the presence of electron correlation in the system [59]. The greater E^ES^ values indicate the better orbital overlapping between hydrogen bond donor and acceptor. Furthermore, the large E^EX^ and E^REP^ terms also imply the feasibility of orbital overlapping between the two monomers. The E^EX^ obtained from GKS-EDA analysis is similar to the charge transfer [60]. The E^POL^ terms are also attractive in nature and such effects are considered by the orbital interactions between monomers which further allows charge shift between the respective monomers [61]. Figure 6 shows a graphical representation of the six components’ contribution (%) in GKS-EDA at the B3LYP-D3/aug-cc-pVTZ level. The E^REP^ term is positive, which is about ~76%–82% of the total attractive energies. The negative values are the attractive components, i.e., E^ES^, E^EX^, E^POL^, E^DISP^, and E^CORR^, and make a large contribution in stabilizing the bimolecular complexes. Among them, the terms E^ES^ and E^EX^ are the dominating contributors, about ~30%–34% and ~44%–46% to the total attractive energies, respectively. E^ES^ and E^EX^ are the main driving forces for the formation of the MeOH complexes. The E^POL^ contribution is about ~11%–14% to the total attractive energies. The E^CORR^ (~4%–5% to the total attractive energies) and E^DISP^ (~4%–9% to the total attractive energies) contributions are close to each other and they are the smallest ones among the five attractive components. The decomposition of the total interaction energy shows that the contribution of E^ES^ and E^EX^ to total interaction energies is very large.

## 3. Computational Methods

All calculations were performed using the Gaussian 09 (Revision E.01) set of programs [62]. Geometry optimizations were carried out using the B3LYP-D3 method with the aug-cc-pVTZ basis set. The B3LYP-D3 functional has been parameterized for describing dispersion interaction, which has been shown to make an important contribution [63]. The harmonic frequencies were calculated at the same level to characterize the stationary points found as minima of the potential energy surface and to evaluate the corresponding zero point vibrational energy (ZPVE). A “very tight” optimization convergence criteria and an “ultrafine” numerical integration grid was used for the DFT calculations to obtain reliable results [64,65]. The binding energies (*BEs*) of the complexes were corrected with ZPVE. However, *BEs* are not corrected for the basis set superposition errors (BSSEs) [66]. The commonly used counterpoise (CP) correction has been found to be important for small basis sets, but not for larger basis sets, such as aug-cc-pVTZ [67,68].

The topological analysis of the electron density, *ρ*(r), and its Laplacian function, ∇^2^*ρ*(r) constitutes a powerful tool to investigate the nature of the chemical bonds [25,26]. According to Bader theory, the presence of a bond critical point (BCP) is a universal indicator of the existence of a bonding interaction [69]. Topological analysis of the charge density was carried out by utilizing the AIM2000 program. The hydrogen bonding interactions were investigated using the generalized Kohn-Sham energy decomposition analysis (GKS-EDA), which is implemented in the GAMESS (US) program [70]. The total interaction energy (E^INT^) is divided into electrostatic energy (E^ES^), exchange energy (E^EX^), repulsion energy (E^REP^), polarization energy (E^POL^), Grimme’s dispersion energy (E^DISP^) and correlation energy (E^CORR^) [27].

## 4. Conclusions

The hydrogen bonded complexes between methanol and amides show that, in the most stable conformation, amides behave simultaneously as a hydrogen bond donor and acceptor. The systematic changes of the substituent make the amides a better hydrogen bond acceptor but a worse hydrogen bond donor. Large red shifts (147–320 cm^−1^) of the OH-stretching transition were found for the MeOH complexes. On the other hand, the red shifts of the NH-stretching transition were much smaller, below 160 cm^−1^. The C=O stretching fundamental transitions were slightly red shifted upon complexation. AIM analyses show the existence of hydrogen bonds in the MeOH complexes, and GKS-EDA analysis demonstrates that the electrostatic, exchange, and repulsion energy play a major role in the total interaction energy.

## Figures and Tables

**Table 1:** Selected optimized geometric parameters in the O–H···O, N–H···O and C=O of the hydrogen bonded MeOH–amide and amide–MeOH complexes at the B3LYP-D3/aug-cc-pVTZ level (angles in degrees; lengths/distances in Å).

Type	Conformer	O–H···O				N–H···O			C=O
		Δr(OH) ^a^	r(HB) ^b^	θ(HB) ^c^	d(O---O) ^d^	Δr(NH) ^e^	r(HB) ^b^	θ(HB) ^c^	Δr(C=O) ^f^
MeOH–amide (A)	FA	0.0102	1.8940	158.3	2.8193	--	--	--	0.0080
	AA	0.0126	1.8491	166.3	2.8043	--	--	--	0.0087
	*cis*-NMF	0.0113	1.8735	160.4	2.8084	--	--	--	0.0087
	*cis*-NMA	0.0134	1.8326	168.0	2.7929	--	--	--	0.0088
	*trans*-NMF	0.0113	1.8715	161.5	2.8103	--	--	--	0.0087
	*trans*-NMA	0.0135	1.8348	167.7	2.7945	--	--	--	0.0091
	DMF	0.0119	1.8614	162.2	2.8035	--	--	--	0.0091
	DMA	0.0136	1.8252	169.5	2.7891	--	--	--	0.0091
MeOH–amide (B)	FA	0.0137	1.8615	153.6	2.7992	0.0088	2.0569	139.5	0.0116
	AA	0.0159	1.8615	153.6	2.7708	0.0089	2.0569	139.5	0.0126
	*cis*-NMF	0.0153	1.8789	152.1	2.7792	0.0085	2.0439	141.1	0.0125
	*cis*-NMA	0.0171	1.8446	154.6	2.7600	0.0084	2.0448	143.8	0.0131
	*trans*-NMF	0.0110	1.8621	174.7	2.8312	--	--	--	0.0079
	*trans*-NMA	0.0121	1.8393	176.6	2.8111	--	--	--	0.0076
	DMF	0.0108	1.8473	170.4	2.8099	--	--	--	0.0070
	DMA	0.0122	1.8357	171.7	2.8021	--	--	--	0.0095
Amide–MeOH	FA	--	--	--	--	0.0049	1.9530	169.5	0.0039
	AA	--	--	--	--	0.0033	1.9908	164.1	0.0031
	*trans*-NMF	--	--	--	--	0.0057	1.9584	169.9	0.0043
	*trans*-NMA	--	--	--	--	0.0055	1.9921	164.4	0.0028

^a^ Δr(OH) = r_complex_ − r_MeOH_, is the change in the OH bond length upon complexation; ^b^ Inter-molecular hydrogen bond distance; ^c^ Inter-molecular hydrogen bond angle; ^d^ Contact distance between the two oxygen atoms in the hydrogen bond (−O−H∙∙∙O=); ^e^ Δr(NH) = r_complex_ − r_amide_, is the change in the NH bond length upon complexation; ^f^ Δr(C=O) = r_complex_ − r_amide_, is the change in the C=O bond length upon complexation; FA: formamide; NMF: *N*-methylformamide; DMF: *N*,*N*-dimethylformamide; AA: acetamide; NMA: *N*-methylacetamide; DMA: *N*,*N*-dimethylacetamide.

**Table 2:** Calculated binding energy (*BE*), zero-point vibrational energy (ZPVE), enthalpy of formation (Δ$H_{298K}^{\theta }$), Gibbs free energy of formation (Δ$G_{298K}^{\theta }$ ) at 298.15 K and 1 atm for the MeOH–amide and amide–MeOH complexes ^a^.

Type	Conformer	*BE* ^b^	ZPVE	Δ$H_{298K}^{\theta }$	Δ$G_{298K}^{\theta }$
MeOH−amide (A)	FA	−23.8	6.0	−23.0	8.9
	AA	−29.0	6.4	−28.7	9.1
	*cis*-NMF	−25.8	5.8	−24.7	7.3
	*cis*-NMA	−30.5	5.9	−29.6	4.6
	*trans*-NMF	−25.7	5.4	−24.4	7.6
	*trans*-NMA	−30.3	6.1	−29.7	10.0
	DMF	−26.7	5.5	−25.4	6.3
	DMA	−30.8	5.8	−29.8	4.4
MeOH−amide (B)	FA	−35.6	8.1	−36.3	2.6
	AA	−37.6	7.9	−38.2	9.1
	*cis*-NMF	−37.9	7.4	−38.2	0.9
	*cis*-NMA	−39.2	7.3	−39.4	2.1
	*trans*-NMF	−29.0	6.2	−28.4	9.6
	*trans*-NMA	−30.1	6.8	−29.9	12.0
	DMF	−27.9	5.7	−26.6	5.3
	DMA	−29.0	6.2	−28.3	8.5
amide–MeOH	FA	−21.2	4.8	−19.5	8.3
	AA	−21.5	5.3	−20.2	13.1
	*trans*-NMF	−22.0	3.7	−19.7	9.8
	*trans*-NMA	−21.9	4.8	−20.3	17.0

^a^ Obtained at the B3LYP-D3/aug-cc-pVTZ level; energies are in kJ·mol^−1^; ^b^ *BEs* are corrected with ZPVE; FA: formamide; NMF: *N*-methylformamide; DMF: *N*,*N*-dimethylformamide; AA: acetamide; NMA: *N*-methylacetamide; DMA: *N*,*N*-dimethylacetamide.

**Table 3:** Calculated bonded OH-, NH- and C=O stretching vibrational wavenumbers and red shifts (in cm^−1^) of the MeOH–amide and amide–MeOH complexes at the B3LYP-D3/aug-cc-pVTZ level.

Type	Conformer	O–H			N–H			C=O		
		$\tilde{\nu }$	Δ$\tilde{\nu }$ ^a^	f_D_/f_M_^b^	$\tilde{\nu }$	Δ$\tilde{\nu }$ ^a^	f_D_/f_M_^b^	$\tilde{\nu }$	Δ$\tilde{\nu }$ ^a^	f_D_/f_M_^b^
MeOH–amide (A)	FA	3623	205	18.5	--	--	--	1758	26	0.9
	AA	3576	252	24.7	--	--	--	1727	28	0.8
	*cis*-NMF	3601	227	20.1	--	--	--	1747	24	0.9
	*cis*-NMA	3560	268	27.5	--	--	--	1718	26	0.8
	*trans*-NMF	3602	226	23.0	--	--	--	1737	28	0.8
	*trans*-NMA	3590	238	24.7	--	--	--	1750	30	0.8
	DMF	3589	238	24.7	--	--	--	1720	27	0.8
	DMA	3557	271	29.0	--	--	--	1683	28	0.7
MeOH–amide (B)	FA	3590	238	24.7	3684 (3452)	28 (122)	2.4 (3.8)	1752	32	1.0
	AA	3527	301	25.0	3695 (3456)	28 (131)	2.3 (1.9)	1721	35	0.9
	*cis*-NMF	3539	288	20.7	3442	149	4.9	1745	27	1.0
	*cis*-NMA	3507	320	29.2	3452	160	0.9	1714	29	1.0
	*trans*-NMF	3610	217	21.0	--	--	--	1742	22	0.8
	*trans*-NMA	3590	238	24.7	--	--	--	1712	23	0.7
	DMF	3681	147	21.9	--	--	--	1731	16	0.8
	DMA	3584	244	22.8	--	--	--	1681	30	0.8
Amide–MeOH	FA	--	--	--	3568 (3487)	53 (87)	3.0 (10.2)	1773	11	0.9
	AA	--	--	--	3675 (3521)	48 (66)	3.1 (7.0)	1746	10	0.9
	*trans*-NMF	--	--	--	3591	35	16.6	1752	12	0.9
	*trans*-NMA	--	--	--	3557	95	11.4	1727	7	0.9

The values in the parentheses are the parameters for the symmetric NH-stretching transitions; ^a^ $∆\tilde{\nu }$ = ${\tilde{\nu }}_{\mathrm{monomer}}$ − ${\tilde{\nu }}_{\mathrm{complex}}$; ^b^ f_D_/f_M_ represents the increase of intensity upon complexation; FA: formamide; NMF: *N*-methylformamide; DMF: *N*,*N*-dimethylformamide; AA: acetamide; NMA: *N*-methylacetamide; DMA: *N*,*N*-dimethylacetamide.

**Table 4:** Atoms in Molecules (AIM) parameters for the MeOH–amide and amide–MeOH complexes obtained at the B3LYP-D3/aug-cc-pVTZ level (distances in Å; all other values in a.u.).

Type	Conformer	O–H···O (C–H···O)				N–H···O (C–H···O)			
		Δ*q*(H) ^a^	*ρ*(BCP) ^b^	∇^2^*ρ*(BCP) ^c^	r ^d^	Δ*q*(H) ^a^	*ρ*(BCP) ^b^	∇^2^*ρ*(BCP) ^c^	r ^d^
MeOH–amide (A)	FA	0.036	0.0230	0.1255	--	--	--	--	--
	AA	0.046	0.0243	0.1419	1.9492	(0.073)	(0.0070)	(0.0329)	(0.9857)
	*cis*-NMF	0.040	0.0238	0.1318	--	--	--	--	--
	*cis*-NMA	0.049	0.0248	0.1478	1.8697	(0.045)	(0.0081)	(0.0359)	(1.0279)
	*trans*-NMF	0.039	0.0239	0.1322	--	--	--	--	--
	*trans*-NMA	0.050	0.0249	0.1462	2.0597	(0.034)	(0.0063)	(0.0300)	(0.7400)
	DMF	0.043	0.0232	0.1433	--	--	--	--	--
	DMA	0.050	0.0252	0.1513	1.8371	(0.031)	(0.0083)	(0.0356)	(0.9545)
MeOH–amide (B)	FA	0.044	0.0226	0.1254	1.6165	0.060	0.0178	0.0939	1.5660
	AA	0.050	0.0244	0.1411	1.6115	0.059	0.0178	0.0922	1.5223
	*cis*-NMF	0.048	0.0238	0.1346	1.6220	0.060	0.0184	0.0932	1.5306
	*cis*-NMA	0.056	0.0251	0.1476	1.5958	0.053	0.0183	0.0939	1.4802
	*trans*-NMF	0.039	0.0239	0.1322	--	--	--	--	--
	*trans*-NMA	0.050	0.0249	0.1462	2.0597	(0.034)	(0.0063)	(0.0300)	(0.7400)
	DMF	0.042	0.0244	0.1369	--	--	--	--	--
	DMA	0.050	0.0252	0.1513	1.8371	(0.031)	(0.0083)	(0.0356)	(0.9545)
Amide–MeOH	FA	--	--	--	--	0.061	0.0204	0.1052	--
	AA	(0.016)	(0.0063)	(0.0225)	(0.8941)	0.055	0.0199	0.1013	1.8910
	*trans*-NMF	--	--	--	--	0.066	0.0204	0.1036	--
	*trans*-NMA	(0.016)	(0.0059)	(0.0210)	(0.7357)	0.052	0.0198	0.1032	1.9016

The values in the parentheses are the AIM parameters for the C−H···O hydrogen bonds; ^a^ The change in atomic charge at the H atom; ^b^ The electron density at the BCPs; ^c^ The Laplacian electron density at the BCPs; ^d^ The distance between a BCP and an RCP; FA: formamide; NMF: *N*-methylformamide; DMF: *N*,*N*-dimethylformamide; AA: acetamide; NMA: *N*-methylacetamide; DMA: *N*,*N*-dimethylacetamide.

**Table 5:** Results of generalized Kohn-Sham energy decomposition analysis (GKS-EDA) for the MeOH–amide and amide–MeOH complexes at the B3LYP-D3/aug-cc-pVTZ level (all values in kJ·mol^−1^) ^a^.

Type	Conformer	ΔE^ES^	ΔE^EX^	ΔE^REP^	ΔE^POL^	ΔE^DISP^	ΔE^CORR^	ΔE^INT^
MeOH–amide (A)	FA	−45.3	−61.1	106.8	−19.2	−5.9	−5.3	−30.1
	AA	−54.4	−77.3	134.8	−22.9	−8.3	−7.7	−35.8
	*cis*-NMF	−48.1	−65.1	113.8	−20.6	−6.1	−5.8	−31.9
	*cis*-NMA	−57.3	−81.8	142.8	−24.5	−7.9	−8.2	−36.8
	*trans*-NMF	−47.7	−64.8	113.4	−20.4	−6.1	−5.9	−31.5
	*trans*-NMA	−56.1	−79.8	139.4	−24.0	−8.6	−7.9	−36.9
	DMF	−49.4	−67.2	117.6	−21.4	−6.3	−6.0	−32.7
	DMA	−58.1	−84.3	147.0	−24.8	−8.4	−8.5	−37.1
MeOH–amide (B)	FA	−74.9	−104.3	183.9	−29.7	−8.3	−11.3	−44.5
	AA	−80.3	−113.8	201.1	−32.5	−8.7	−12.5	−46.6
	*cis*-NMF	−79.2	−112.5	198.6	−31.8	−8.8	−12.5	−46.3
	*cis*-NMA	−83.8	−121.4	214.5	−34.0	−9.3	−13.6	−47.5
	*trans*-NMF	−51.6	−73.7	127.8	−21.0	−9.5	−7.6	−35.5
	*trans*-NMA	−54.4	−77.7	135.0	−22.5	−9.7	−7.7	−37.1
	DMF	−53.1	−76.5	133.0	−22.0	−8.2	−8.1	−34.8
	DMA	−53.1	−78.2	136.0	−22.6	−10.2	−8.7	−36.8
Amide–MeOH	FA	−36.3	−46.5	81.1	−14.3	−6.1	−3.8	−26.0
	AA	−34.8	−50.7	85.9	−12.6	−9.9	−4.5	−26.6
	*trans*-NMF	−35.6	−47.6	82.6	−13.8	−7.1	−4.2	−25.6
	*trans*-NMA	−34.3	−51.9	87.7	−12.3	−10.8	−4.9	−26.4

a The total interaction energy (ΔE^INT^) is divided into electrostatic energy (ΔE^ES^), exchange energy (ΔE^EX^), repulsion energy (ΔE^REP^), polarization energy (ΔE^POL^), Grimme’s dispersion energy (ΔE^DISP^) and correlation energy (ΔE^CORR^); FA: formamide; NMF: *N*-methylformamide; DMF: *N*,*N*-dimethylformamide; AA: acetamide; NMA: *N*-methylacetamide; DMA: *N*,*N*-dimethylacetamide.
